# Research Quality—Lessons from the UK Research Excellence Framework (REF) 2021

**DOI:** 10.3390/nursrep12030048

**Published:** 2022-07-07

**Authors:** David R. Thompson, Hugh P. McKenna

**Affiliations:** 1 School of Nursing and Midwifery, Queen’s University Belfast, Belfast BT7 1NN, UK; 2 School of Nursing, Ulster University, Newtownabbey BT37 0QB, UK

Research quality is a term often bandied around but rarely clearly defined or measured. Certainly, the nursing contribution to research and research quality has often been under-recognised, under-valued and under-funded, at least in the UK. For over 20 years, it has been argued that there should be more investment in, and acknowledgement of, nursing’s contribution to high-quality research [1,2].

One way of measuring the quality of research in nursing across different higher education institutions (HEIs) and its parity with other disciplines is through periodic national research assessment exercises. In 1986, the first national Research Assessment Exercise (RAE) in higher education (HE) took place in the UK under the government of Margaret Thatcher. The purpose of the exercise was to determine the allocation of funding to UK universities at a time of tight budgetary restrictions. Since then, RAEs took place in 1989, 1992, 1996, 2001 and 2008 in its different iterations. In response to criticisms, the scale and assessment process has changed markedly since the first RAE.

## 1. The Research Excellence Framework (REF)

In 2014, the first Research Excellence Framework (REF) replaced the RAE. In 2016, the Stern report [3] identified five purposes for the REF:

- To provide accountability for public investment in research and produce evidence of the benefits of this investment.
- To provide benchmarking information and reputational yardsticks for the HE sector and for public information.
- To provide a rich evidence base to inform strategic decisions about national research priorities.
- To create strong performance incentives for HEIs/researchers.
- To inform decisions on the selective allocation of non-hypothecated funding for research.

The REF2021 has just reported its assessment and its outcomes inform the research grant allocations from the four HE funding bodies, with effect from 2022–2023. This quality related (QR) research funding, totalling £2 billion per year, enables HEIs to conduct their own directed research, much of which is supported subsequently by the UKRI Research Councils and other bodies (charities, industry, EU, etc.): known as the Dual Support System. So, in essence, this is an important exercise for universities and their disciplines in terms of funding, but also reputation, image and prestige.

The REF2021, for the first time, included the submission of all staff with significant responsibility for research. A total of 157 UK HEIs participated, submitting over 76,000 academic staff. As the REF is a discipline-based expert review process, 34 expert sub-panels, working under the guidance of four main panels, reviewed the submissions and made judgements on their quality. The panels comprised 900 academics, including 38 international members, and 220 users of research.

The rigour, significance and originality of research outputs (185,594) were judged on a 5-point scale:

- 4* (quality that is world-leading in terms of originality, significance and rigour);
- 3* (quality that is internationally excellent in terms of originality, significance and rigour but which falls short of the highest standards);
- 2* (quality that is recognised internationally in terms of originality, significance and rigour);
- 1* (quality that is recognised nationally in terms of originality, significance and rigour);
- Unclassified (below the quality threshold for 1* or does not meet the definition of research used for the REF).

Impact case studies (6781) were assessed in terms of reach and significance of impacts on the economy, society and/or culture [4].

Environment was assessed in terms of vitality and sustainability (strategy, people, income and collaboration).

Results were produced as “overall quality profiles”, which show the proportions of submitted activity judged to have met each quality level from 4* to unclassified. Submissions included research outputs, examples of the impact and wider benefits of research and evidence about the research environment. The overall quality profile awarded to each submission is derived from three elements that were assessed: the quality of research outputs (contributing 60% of the profile); the social, economic and cultural impact of research (contributing 25% of the profile); and the research environment (contributing 15%) of the overall quality profile. The panels reviewed the submitted environment statements and statistical data on research income and doctoral degrees. A statement about the overall institution’s environment was provided to inform and contextualise the panel’s assessment.

Key findings were that the overall quality was 41 per cent world-leading (4*) and 43 per cent internationally excellent (3*) across all submitted research activities. At least 15 per cent of the research was considered world-leading (4*) in three-quarters of the UK’s HEIs. In terms of impacts, the expert panels observed the significant gains made from university investment in realising research impact. However, changes between the REF2014 and REF2021 exercises limit the extent to which meaningful comparisons can be made across results, particularly outputs.

## 2. How Did Nursing Fare?

Nursing research was primarily, but not exclusively, submitted to the REF2021 sub-panel (Unit of Assessment) 3 (Allied Health Professions, Dentistry, Nursing and Pharmacy). SP3 received 89 submissions from 90 universities covering a very wide range of disciplines, thus making it difficult to focus specifically on research emanating from the discipline of nursing. Additionally, some research on nursing-related issues and interventions may have been included in returns to other units of assessment and not seen by SP3 members.

Nevertheless, some overall impressions of nursing emerged:

### 2.1. Strengths

There was evidence of a strong level of interdisciplinary collaboration and examples of strong academic nursing leadership in large multidisciplinary research teams. The scale of research activity in nursing ranged from modest or emerging centres of nursing research through to substantial, long-established units with mature research environments. A notable feature was high quality research addressing a wide range of nursing-related issues of critical importance to recipients of nursing care.

Many of the strongest outputs focused on people’s quality of life and health outcomes and included interventions designed to support older people and those with enduring health challenges, including symptom management, self-management and managing continence, mobility problems and pain. There were also signs of a growing emphasis on evaluating new approaches to care delivery and new or extended roles which aim to enhance access to care.

There were outstanding case studies that demonstrated clear links to the underpinning research related to the outputs, including in mental health, ageing, dementia, enduring health challenges and self-management of care. There was clear evidence of impact and reach on society, policy, practice and the economy, including changes to public perceptions of health. Evidence of reach was demonstrated through improvements to healthcare practice and delivery which enhanced health outcomes and quality of life.

There was evidence of highly developed research environments in which a significant volume of world leading or internationally excellent quality research was being generated within the nursing discipline. There was evidence of nurses leading large research centres and institutions where mature inter- and trans-disciplinary research was facilitated with a clear and joined up strategy. Most stronger submissions demonstrated clear institutional strategic commitment to and investment in furthering research in the discipline. There was also evidence of methodological developments, strong collaborations with non-academic partners, explicit attention to equality, diversity and inclusion and support for early career researchers. There was good evidence of national and international esteem demonstrated in the discipline through journal editorships, grant awards panel membership, significant positions in national institutions as well as some examples of national honours and other forms of recognition.

The early research assessment exercises were instrumental in sustaining research funding in elite universities and it achieved that goal for decades. Many of these had no nursing departments (e.g., Oxford, Cambridge, Imperial). However, the results of REF2021 show that this is no longer the case. In REF2021, the “golden triangle” universities have lost 2.4 percentage points and there appears to be a “levelling-up” of research with islands of excellence across the UK, often in universities with large nursing departments. For example, Northumbria leaped from 52 to 28 places in the market share table for QR funding. Similar trends were seen at Manchester Metropolitan University (56 to 38) and Portsmouth (60 to 47).

### 2.2. Challenges

It was sometimes difficult to determine how research activity in nursing was organised, supported and resourced. This included a few stronger research-led universities where there was little evidence that nursing was being prioritised equitably with other disciplines in terms of supporting growth and sustainability.

There were some outputs that were iterative scoping and systematic reviews which did not generate new knowledge and had difficulty meeting the originality and significance criteria. Furthermore, we know that nurses are undertaking excellent pedagogic research, yet little or none of this was returned to SP3, even though the panel’s descriptor showed that it would be welcome. Perhaps it was submitted to other expert panels such as SP23 (Education). Similarly, SP3 received few public engagement impact case studies, even though we know that nurses undertake excellent research in partnership with the public and patients.

It was apparent that, in general, nursing appears to have gained limited access to major funding schemes beyond the National Institute for Health and Care Research (NIHR) compared with many of the other disciplines included in UoA3. Additionally, there was a paucity of career development and personal award schemes to support post-doctoral nurses, which may be a key factor influencing the overall low numbers of early career researchers included in the submissions. Another concern was the low percentage of eligible nursing staff returned by some institutions, which raises the question of the extent to which eligible nursing staff in these institutions are being given time and support to undertake research as part of their academic role. This was often accompanied by a low number of early career researchers, down 3% from the REF2014 exercise, and none returned by some nursing departments. The combination of lower early career researcher numbers with a low return of eligible staff in some universities raises questions about research capacity and capability support.

These issues suggest strongly that there needs to be significant investment in nursing and more attention to the research priorities of major grant awarding bodies and access to a broader base of research funding opportunities. Mentorship, coaching and support will also promote the development of research leaders and strengthen the growth and spread of high-quality research activity.

A major and enduring challenge for nursing has been building and sustaining research capacity and capability. Nursing has long suffered from a lack of proper investment, including funding, and failure to influence the priorities of research funders. These are issues that need to be addressed urgently, particularly as there remain concerns about the availability of nurses in universities and health services at all levels, but particularly early career researchers, in the pipeline [5,6]. National strategies for nursing research have long been articulated [1], but little progress has been made in their implementation. Such strategies need to raise the profile of nursing research, ensure it is valued and that nurses in universities and the health services have protected research time and access to the same level of funding and support as their peers in other disciplines.

A number of expert committees have been established to identify how the REF structure and process can be improved prior to the next iteration in 2028. Sir Peter Gluckman is chairing a group of international experts and they report in October 2022. There are also committees examining the use of quantitative metrics and artificial intelligence (AI) in research assessment. What is clear is that peer review will remain, but informed by AI, metrics, and citations. Another emerging theme is the greater use of qualitative data and an emphasis on research culture, both of which should benefit nursing.

## 3. Conclusions

Research assessment exercises in one form or another seem likely to stay. Though imperfect, they are an important, perhaps the best available, means of ensuring scrutiny and accountability of public investment in research and for benchmarking disciplines and universities. However, the REF is merely the tip of the iceberg regarding the research and impact of UK nursing. Therefore, we should not let the REF tail wag the research dog. It is important to rejoice in the improved outcomes of nursing, but all contributions should be celebrated. Many nurses have expertly undertaken heavy administration and teaching loads so that the very best research can be submitted.

The REF is important because such exercises, beginning in the UK in 1986, have been emulated or adapted by other countries across the world, including Australia, New Zealand, Hong Kong, The Netherlands, Germany, Italy, Romania, Denmark, Finland, Norway, Sweden and the Czech Republic. The results inform the allocation of public funding on which the viability and reputation of research in nursing depends. One of the criticisms of the RAE was that university presidents used poor ratings as a reason to close, cut or merge departments/disciplines. Nursing can ill-afford to be at the mercy of such decision making. It is incumbent on the discipline to demonstrate forcibly the quality and impact of its research on the health and wellbeing of the people and communities it serves.
